# Epigenetic features of *FoxP3* in children with cow’s milk allergy

**DOI:** 10.1186/s13148-016-0252-z

**Published:** 2016-08-12

**Authors:** Lorella Paparo, Rita Nocerino, Linda Cosenza, Rosita Aitoro, Valeria D’Argenio, Valentina Del Monaco, Carmen Di Scala, Antonio Amoroso, Margherita Di Costanzo, Francesco Salvatore, Roberto Berni Canani

**Affiliations:** 1 Department of Translational Medical Science, University of Naples “Federico II”, Via S. Pansini, 5 80131 Naples, Italy; 2 CEINGE-Biotecnologie Avanzate s.c.ar.l, Via Gaetano Salvatore 486, 80131 Naples, Italy; 3 Department of Molecular Medicine and Medical Biotechnologies, University of Naples Federico II, Via S. Pansini 5, 80131 Naples, Italy; 4 IRCCS-Fondazione SDN, Via E. Gianturco 113, 80143 Naples, Italy; 5 European Laboratory for the Investigation of Food-Induced Diseases, University of Naples “Federico II”, Via S. Pansini 5, 80131 Naples, Italy

**Keywords:** Food allergy, Extensively hydrolyzed casein formula, Oral tolerance, *Lactobacillus rhamnosus* GG

## Abstract

**Background:**

DNA methylation of the Th1 and Th2 cytokine genes is altered during cow’s milk allergy (CMA). Forkhead box transcription factor 3 (*FoxP3*) is essential for the development and function of regulatory T cells (Tregs) and is involved in oral tolerance acquisition. We assessed whether tolerance acquisition in children with IgE-mediated CMA is associated with DNA demethylation of the Treg-specific demethylated region (TSDR) of *FoxP3*.

**Results:**

Forty children (aged 3–18 months) were enrolled: 10 children with active IgE-mediated CMA (group 1), 10 children who outgrew CMA after dietary treatment with an extensively hydrolyzed casein formula containing the probiotic *Lactobacillus rhamnosus* GG (group 2), 10 children who outgrew CMA after treatment with other formulas (group 3), and 10 healthy controls (group 4). *FoxP3* TSDR demethylation and expression were measured in mononuclear cells purified from peripheral blood of the four groups of children. *FoxP3* TSDR demethylation was significantly lower in children with active IgE-mediated CMA than in either children who outgrew CMA or in healthy children. Formula selection influenced the *FoxP3* TSDR demethylation profile. The *FoxP3* TSDR demethylation rate and expression level were correlated.

**Conclusions:**

Tolerance acquisition in children with IgE-mediated CMA involves epigenetic regulation of the *FoxP3* gene. This feature could be a new target for preventive and therapeutic strategies against CMA.

## Background

Epigenetic mechanisms have been implicated in the pathogenesis of food allergy [1]. We previously demonstrated that tolerance acquisition in children with IgE-mediated cow’s milk allergy (CMA) is driven by epigenetic modulation of the Th1 and Th2 cytokine genes [2]. A regulatory T cell (Treg) suppressive phenotype, characterized by stable expression of the transcription factor “Forkhead box Protein 3” (*FoxP3*), plays a pivotal role in food tolerance [3–7]. *FoxP3* messenger RNA (mRNA) expression is lower in children with atopic asthma or IgE-mediated food allergy than in healthy children [8]. *FoxP3* stable expression requires full CpG demethylation of its transcriptional regulatory regions [9, 10], and, moreover, hypermethylation of the *FoxP3* gene has been associated with reduced Treg function and allergy [7, 11].

DNA methylation is a biologically and chemically stable epigenetic modification that locks in long-term gene expression patterns [12, 13]. The demethylation status of *FoxP3* at a highly conserved region within the Treg-specific demethylated region (TSDR), a CpG-rich region, located on the 2nd conserved non-coding sequence of *FoxP3* (CNS2), is restricted to Tregs [14, 15]. Transcriptional activity of the TSDR is essentially determined by its methylation status: it is completely inactive in its methylated state, but when the TSDR is demethylated, transcription factors such as Ets-1 and Creb can bind to the TSDR [16] TSDR demethylated and open chromatin conformation in the *Foxp3* locus leads to stable phenotype differentiated Foxp3+ Treg [17, 18]. *FoxP3* TSDR demethylation in peripheral blood mononuclear cells (PBMCs) has been associated with reduced atopic sensitization and asthma in children [19]. Epigenetic regulation of antigen-induced T cell subsets may predict a state of immune tolerance in food allergy. Indeed, DNA methylation of the *FoxP3* gene in Tregs decreased during oral tolerance acquisition in patients with peanut allergy undergoing oral immunotherapy [19]. The aim of this study was to evaluate further the epigenetic regulation of *FoxP3* gene in children with IgE-mediated CMA.

## Methods

### Study subjects

We evaluated IgE-mediated CMA children (aged 3 to 18 months) consecutively referred to our tertiary Pediatric Allergy Center for oral food challenge. Oral food challenge was requested to obtain a diagnosis because of recent evidence of CMA signs and symptoms (“active CMA patients”; group 1) or to investigate the occurrence of oral tolerance acquisition after dietary treatment with an extensively hydrolyzed casein formula containing the probiotic *Lactobacillus rhamnosus* GG (LGG, 1 × 10^6^/ml CFU) (“subjects who outgrew CMA with extensively hydrolyzed casein formula (EHCF) + LGG”; group 2) or with other formulas (“subjects who outgrew CMA with other formulas”; group 3). All patients underwent a double-blind placebo-controlled oral challenge (DBPCFC), as described previously [20]. All oral food challenges took place at our Center on two separate days with a 1-week interval. Parents of children taking antihistamine were advised to withhold these medications for at least 72 h before and during the challenge. Randomization and preparation of the challenges were performed by experienced food allergy dieticians not directly involved in the procedures. Briefly, every 20 min, successive doses (0.1, 0.3, 1, 3, 10, 30, and 100 ml) of fresh pasteurized cow’s milk containing 3.5 % fat (verum) or hypoallernic or soy formula that the child was already consuming (placebo) were administered. Full emergency equipment and medications (epinephrine, antihistamines, and steroids) were available. The results were assessed simultaneously by three experienced pediatric allergists. Study subjects were scored for nine items divided into four main categories: (i) general (lowered blood pressure plus tachycardia); (ii) skin (rash, urticaria/angioedema); (iii) gastrointestinal (nausea/repeated vomiting, crampy-like abdominal pain, diarrhea); and (iv) respiratory (sneezing/itching, nasal congestion/rhinorrhea, stridor deriving from upper airway obstruction or wheezing) on a 0- to 3-point scale (0, none; 1, light; 2, moderate; and 3, severe). If at least two of the three physicians independently scored any item at level 3, or 2 (or more) items at level 2, the test result was considered positive. Clinical symptoms occurring within 2 h of administering the highest dose were defined as “IgE-mediated reactions.” The infants were observed for 2 h after the final dose and then discharged. In the case of a positive DBPCFC, at any testing dose, the patient remained under observation until symptom resolution. If the patient did not show any symptom within the first 24 h, parents were advised to give one single feed of 100 ml of the tested formula (verum or placebo) everyday at home for 7 days. If any symptom occurred during this period, the patient returned to the outpatient clinic on the same day. After 7 days of verum or placebo administration, the patients were examined and the parents interviewed at the Center. To rule out a false-negative challenge result, parents were asked to contact the Center if any symptoms occurred in the 7 days after the DBPCFC procedures. The challenge was considered negative if the patient tolerated the entire challenge, including the observation period. Clinical tolerance acquisition was defined by the presence of a negative DBPCFC. Patients with a negative oral challenge (groups 2 and 3) were reassessed 4 weeks later to verify persistence of clinical tolerance. A venous blood sample (4 ml) was obtained from all patients after oral challenge.

Exclusion criteria were as follows: allergic disorders or food allergies other than CMA, eosinophilic disorders of the gastrointestinal tract, food protein-induced enterocolitis syndrome, concomitant chronic systemic diseases, congenital cardiac defects, active tuberculosis, autoimmune diseases, immunodeficiency, chronic inflammatory bowel diseases, celiac disease, cystic fibrosis, metabolic diseases, lactose intolerance, malignancy, chronic pulmonary diseases, and malformations of the gastrointestinal tract. During the same study period, consecutive healthy children, not at risk of atopic disorders (namely, those without a first-degree relative affected by an atopic disorder), attending our Center because of minimal surgical procedures served as a control group (group 4).

A venous blood sample (4 ml) was collected also from these healthy subjects. They were assessed for the presence of food allergy and other allergic diseases at enrollment and 6 months after blood sampling by pediatric allergists at our Center.

### Total IgE and specific IgE against proteins and epitopes of cow’s milk

Serum was obtained by centrifugation for 10 to 15 min. Serum was flash frozen and stored at −80 °C until analysis. Serum total IgE and specific IgE against epitopes of cow’s milk (alpha-lactalbumin, beta-lactoglobulin, bovine serum albumin, casein, lactoferrin) were analyzed by enzymatic immunoassay (Phadia 100 ThermoFisher Scientific CAP system, Rodano Milano, Italy). Results were expressed as kilounits per liter (kU/l).

### DNA methylation and mRNA expression

Peripheral blood mononuclear cells were isolated from whole blood samples using the Ficoll-Paque (Sigma-Aldrich, St. Louis, MO, USA) method, as described previously [2]. The primers used for DNA methylation analysis of *FoxP3* TSDR are reported elsewhere [15]. High-resolution melting real-time PCR for methylation analysis was performed as described previously [2]. The results of methylation analysis were verified by direct sequencing (using the Sanger method modified as follows: ddNTPs labeled with four different fluorophores) and analyzed by capillary electrophoresis (the analytical specificity and sensitivity of the test was >99 %). Real-time PCR was performed with the LightCycler® 480 instrument (Roche Applied Science, Penzberg, Germany) using 96-well plates (Roche Applied Science). Briefly, RNA was extracted from the PBMCs of the four study groups using the Trizol protocol (Invitrogen, Life Technologies Europe BV, Monza, Italy), as previously described [2]. The concentration and purity of RNA samples were measured and verified by NanoDrop 1000 spectrophotometry (Thermo Scientific, Wilmington, DE, USA). For complementary (cDNA) synthesis, 1 μg total RNA was transcribed with a High Capacity cDNA Reverse Transcription kit (Applied Biosystems, Foster City, CA, USA) according to the manufacturer’s instructions. The 10-μl reaction volumes contained 1 μl template, 10 μl SYBR Green (Applied Biosystems), and 5 μM primers (*FoxP3* forward primer 5′-AGCTGGAGTTCCGCAAGAAAC-3′; *FoxP3* reverse primer 5′-TGTTCGTCCATCCTCCTTTCC-3′; GenBank Accession number NC_000023.11). Quantitative real-time amplifications were performed in triplicate with an initial incubation at 95 °C for 30 s, followed by 40 cycles of 95 °C for 10 s and 60 °C for 30 s, using a Light Cycler 79 HT (Applied Biosystems). The quantitative gene expression was calculated with the comparative Ct method and normalized against the Ct of glucuronidase (*GUS*) messenger as reference gene.

### Statistical analysis

The Kolmogorov-Smirnov test was used to determine whether variables were normally distributed. The *χ*^2^ test and Fisher’s exact test were used for categorical variables. We used the *t* test and one-way ANOVA to evaluate differences among continuous variables. To determine which groups in the sample differ, the Bonferroni correction was performed. Pearson’s correlation coefficient “*r*” was used to evaluate the correlation between continuous variables. The level of significance for all statistical tests was two-sided, *P* < 0.05. All analyses were conducted by a statistician blinded to patient group assignment, using SPSS, version 19.0 for Windows (SPSS Inc., Chicago, IL, USA).

## Results

The main demographic and clinical characteristics of the study population are reported in Table 1. The 10 children affected by CMA in group 1 had a positive DBPCFC at doses between 3 and 10 ml of cow’s milk. The 20 CMA children with recent evidence of oral tolerance acquisition, demonstrated by a negative DBPCFC (groups 2 and 3), were able to consume at least one full cup/day of cow’s milk without symptoms. Group 2 patients received EHCF + LGG-based treatment, and group 3 patients received the following formulas: EHCF (*n* = 3), soy formula (SF, *n* = 3), amino acid-based formula (AAF, *n* = 3), hydrolyzed rice formula (RHF, *n* = 1). The median duration of dietotherapy was 384 days (IQR 7.75) in both groups.

**Table 1:** The main demographic and clinical characteristics of the four study groups

	Subjects with CMA at diagnosis	Subjects outgrown CMA		Healthy subjects
		EHCF + LGG	Other formulas	
	Group 1	Group 2	Group 3	Group 4
Number	10	10	10	10
Male, number (%)	7 (70)	7 (70)	6 (60)	4 (40)
Age, months (SD)	5.5 (0.7)	16.9 (0.9)	17 (0.9)	9 (4)
Body weight, kg (SD)	7.385 (964.7)	12.047 (682.4)	12.273 (776.7)	8.075 (3193.5)
Spontaneous delivery, number (%)	5 (50)	2 (20)	3 (30)	4 (40)
Breastfeeding, ≤8 weeks, number (%)	10 (100)	10 (100)	10 (100)	10 (100)
Symptoms at the CMA onset				
Gastrointestinal, *n* (%)	4 (40)	5 (50)	3 (30)	–
Cutaneous, *n* (%)	8 (80)	8 (80)	7 (70)	–
Respiratory, *n* (%)	1 (10)	3 (30)	3 (30)	–
Total serum IgE, kU/l (SD)	260.6 (230.9)	255.9 (236.1)	130.4 (167.9)	0.2 (0.1)
Alpha-lactalbumin, kUA/l(SD)	6 (11.2)	0.6 (0.7)	3.1 (3.9)	–
Beta-lactoglobulin, kUA/l(SD)	4.5 (7.1)	2.2 (3.4)	1.9 (2.6)	–
Bovine serum albumin, kUA/l(SD)	5.4 (8.7)	0.9 (1.8)	1.9 (3)	–
nBCasein, kUA/l(SD)	22.7 (39.2)	0.5 (0.4)	1.3 (2.7)	–
Lactoferrin, kUA/l(SD)	2 (6.1)	0	0	–

**Fig. 1:**
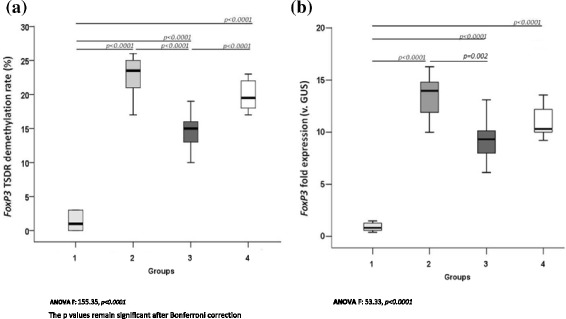
*FoxP3* TSDR demethylation rate (%) (**a**) and *FoxP3* mRNA expression versus GUS expression (**b**) observed in study groups (group 1, CMA children at diagnosis; group 2, children outgrown CMA after treatment with EHCF + LGG; group 3, children outgrown CMA after treatment with other formulas; group 4, healthy controls)

**Fig. 2:**
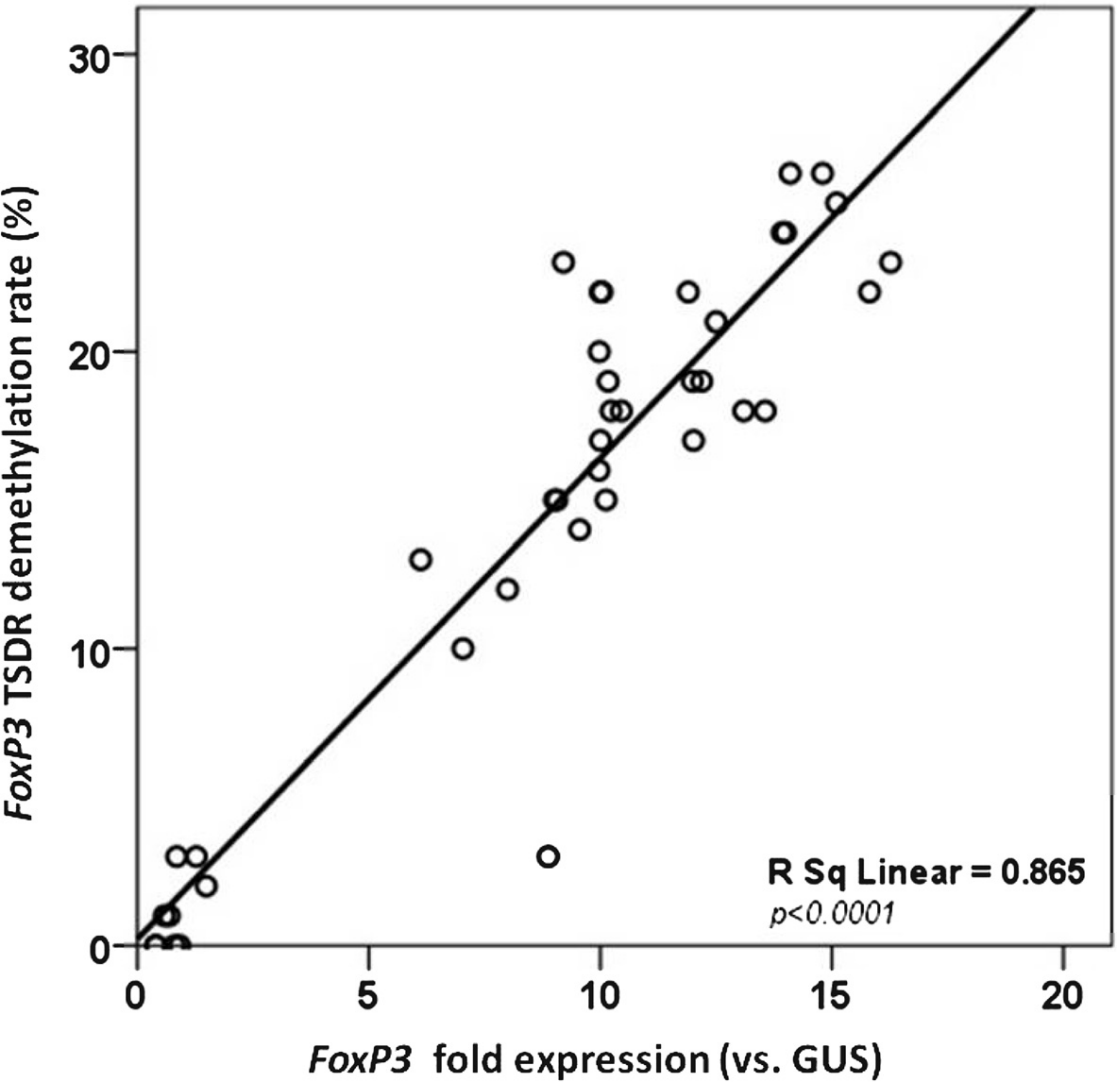
Linear regression analysis of % *FoxP3* TSDR demethylation rate (%) in PBMCs versus *FoxP3* mRNA expression in study subjects

As shown in Fig. 1a, the rate of *FoxP3* TSDR demethylation was lower in active IgE-mediated CMA patients (group 1) than in healthy controls (1.3 ± 1.3 vs 20 ± 2.1, *P* < 0.0001). To determine the fold expression levels, we normalized *FoxP3* TSDR expression vs the transcription levels of the housekeeping gene *GUS* in the four study groups. As shown in Fig. 1b, *FoxP3* expression was significantly lower (*P <* 0.0001) in active IgE-mediated CMA children than in healthy controls. Moreover, it was higher in children who outgrew CMA than in children with active IgE-mediated CMA, but similar to levels in the healthy children. The *FoxP3* TSDR demethylation rate was correlated with *FoxP3* expression levels (Fig. 2). Notably, the *FoxP3* TSDR demethylation profile stratified CMA patients according to disease state. In fact, the demethylation profile in subjects with recent evidence of tolerance acquisition was different to that of active CMA patients and differed significantly (*P <* 0.0001) between children treated with EHCF + LGG and children receiving other formulas (Fig. 1a).

## Discussion

Our data show that tolerance acquisition in children with IgE-mediated CMA involves epigenetic regulation of the *FoxP3* gene. We found that *FoxP3* TSDR demethylation is significantly increased in CMA children who acquired oral tolerance. Similarly, it has been demonstrated that farm milk exposure in children has an allergy preventive effect which is linked to *FoxP3* TSDR demethylation in peripheral blood cells [19].

We found a significant difference in the methylation status of *FoxP3* TSDR gene comparing patients who acquired oral tolerance after treatment with EHCF + LGG vs children treated with other formulas. Similar results have been observed investigating Th1/Th2 cytokines [2]. A recent study showed that LGG in addition to vitamin D supplementation increased the number of CD4^+^CD25^+^FoxP3^+^ Treg in children with allergic rhinitis [21].

We demonstrated that EHCF + LGG but not EHCF alone is able to positively shape gut microbiota composition increasing the abundance of butyrate-producer bacteria strains [22]. Butyrate is able to inhibit histone deacetylase 9 and 6 with subsequent demethylation of *Foxp3* gene [23]. Finally, a formula-induced regulation of circulating miRNAs (microRNA-155, microRNA-148a, microRNA-29b, microRNA-21) acting on Treg differentiation may be also involved [24].

Although with various caveats (the use of PBMCs, a cross-sectional design, and the relatively low number of subjects), our study sheds light on how variations in the level of *FoxP3* TSDR demethylation can affect the course of CMA and demonstrates that dietary intervention influences this epigenetic mechanism. Our results also suggest that the *FoxP3* TSDR demethylation rate could serve as a biomarker of oral tolerance. The novel data reported herein may serve as a basis for the development of new diagnostic and therapeutic tools for CMA.

## Abbreviations

TSDR, Treg-specific demethylated region; AAF, amino acid-based formula; CMA, cow’s milk allergy; EHCF, extensively hydrolyzed casein formula; EHCF + LGG, extensively hydrolyzed casein formula plus *Lactobacillus rhamnosus* GG; FoxP3, forkhead box protein 3; IgE, immunoglobulin E; LGG, *Lactobacillus rhamnosus* GG; PBMCs, peripheral blood mononuclear cells; RHF, hydrolyzed rice formula; SF, soy formula; SPT, skin prick testing; Treg, regulatory T cells
